# A simple preparation step to remove excess liquid lipids in white adipose tissue enabling improved detection of metabolites via MALDI-FTICR imaging MS

**DOI:** 10.1007/s00418-022-02088-y

**Published:** 2022-04-07

**Authors:** Qian Wang, Na Sun, Thomas Kunzke, Achim Buck, Jian Shen, Verena M. Prade, Barbara Stöckl, Jun Wang, Annette Feuchtinger, Axel Walch

**Affiliations:** Research Unit Analytical Pathology, Helmholtz Zentrum München - German Research Center for Environmental Health, Ingolstaedter Landstrasse 1, 85764 Neuherberg, Germany

**Keywords:** White adipose tissue, Layer of excess liquid lipids, Filter paper, MALDI-FTICR imaging MS, Metabolite

## Abstract

Matrix-assisted laser desorption ionization (MALDI) Fourier transform ion cyclotron resonance (FTICR) imaging mass spectrometry (MS) is a powerful technology used to analyze metabolites in various tissues. However, it faces significant challenges in studying adipose tissues. Poor matrix distribution and crystallization caused by excess liquid lipids on the surface of tissue sections hamper *m/z* species detection, an adverse effect that particularly presents in lipid-rich white adipose tissue (WAT). In this study, we integrated a simple and low-cost preparation step into the existing MALDI-FTICR imaging MS pipeline. The new method—referred to as filter paper application—is characterized by an easy sample handling and high reproducibility. The aforementioned filter paper is placed onto the tissue prior to matrix application in order to remove the layer of excess liquid lipids. Consequently, MALDI-FTICR imaging MS detection was significantly improved, resulting in a higher number of detected *m/z* species and higher ion intensities. After analyzing various durations of filter paper application, 30 s was found to be optimal, resulting in the detection of more than 3700 *m/z* species. Apart from the most common lipids found in WAT, other molecules involved in various metabolic pathways were detected, including nucleotides, carbohydrates, and amino acids. Our study is the first to propose a solution to a specific limitation of MALDI-FTICR imaging MS in investigating lipid-rich WAT. The filter paper approach can be performed quickly and is particularly effective for achieving uniform matrix distribution on fresh frozen WAT while maintaining tissue integrity. It thus helps to gain insight into the metabolism in WAT.

**Supplementary Information:**

The online version contains supplementary material available at 10.1007/s00418-022-02088-y.

## Introduction

Adipose tissue, as an endocrine organ with high metabolic activity, consists of adipocytes embracing triglycerides, which are known as the main energy reserves of humans and animals. White adipose tissue (WAT) is the most abundant tissue and predominant adipose type in the body. It plays a key role in the formation of triacylglycerols and the release of fatty acids into the blood when there is a lack of energy (Mercader et al. 2006). WAT cells generally contain a single huge lipid droplet surrounded by a thin layer of cytoplasm, which has squeezed the nucleus, known as the peripheral nucleus, into the rim in cells. Adipose tissue is the primary mediator of metabolism and the site of onset and progression of metabolic diseases. The most common adipose dysfunction disease is obesity. It has been confirmed that obesity-related diseases involve pathological changes mainly in WAT, energy reserve, and endocrine organs (Scherer 2019). To prevent obesity and metabolic disorders, it is crucial to suppress immoderate WAT. However, it is difficult to comprehensively profile the metabolism of WAT owing to its specific histological features, resulting in a layer of excess liquid lipids on WAT sections at room temperature. This adverse effect limits various in situ analyses including staining procedures as well as imaging mass spectrometry (MS).

Matrix-assisted laser desorption ionization (MALDI) Fourier transform ion cyclotron resonance (FTICR) imaging MS is a powerful tool currently used to study metabolomics from fresh frozen tissue samples (Aichler and Walch 2015; Norris and Caprioli 2013). It is useful in biomarker research and molecular tissue classification (Prade et al. 2020; Prentice et al. 2019; Hochart et al. 2019; Maus et al. 2020). With spatial resolution and molecular specificity, MALDI-FTICR imaging MS is valuable for the label-free detection of diverse metabolites (Karas and Hillenkamp 1988; Liu et al. 2018; Kompauer et al. 2017; Beaufour et al. 2018), including lipids (Liu et al. 2021; Wang 2021; Wang et al. 2021). Metabolite profiling in adipose tissue-related diseases has been reported in several publications, albeit using other technologies (Chondronikola et al. 2016; Caesar et al. 2010; Duarte et al. 2014; Pietiläinen et al. 2011). MALDI-FTICR imaging MS has limitations in the analysis of adipose tissue samples, particularly lipid-rich WAT. Many researchers are working to improve MALDI-FTICR imaging MS’s workflow to address specific analytical challenges in WAT. Fernández-Vega et al. (2020) used a special slide covered with carbon conductive tape to avoid lipid dislocalization and investigated adipose tissue-associated disorders. Popkova and Schiller added CsCl to the MALDI matrix solution to overcome ion suppression effects on the mass spectrum and raise the detectability of other invisible or weak phospholipid ions in adipose tissue (Popkova and Schiller 2017).

In contrast to previous studies, we focused on the issue that excess liquid lipids on the surface of WAT sections hamper MALDI matrix deposition, which has so far not been addressed, and still lacks an effective solution. As a critical step during MALDI-FTICR imaging MS workflow, matrix deposition was significantly hampered by the liquid lipid layer, diminishing ionization and limiting quality and quantity of mass spectrum data of WAT. We increased the efficiency of MALDI-FTICR imaging MS in studying adipose tissues, particularly WAT, using filter paper. The results demonstrated that the use of filter paper reduced the adverse effects of the excess liquid lipid layer on MALDI-FTICR imaging MS, allowing improved metabolite detection in WAT.

## Materials and methods

### Tissue preparation and sectioning

WAT was collected from three mice and liver tissue was collected from one mouse. All tissue samples were flash-frozen and preserved in liquid nitrogen until measurement, to keep the tissue shape intact, as well as minimize degradation (Crecelius et al. 2008). All animal studies were conducted following German animal welfare regulations and approved by the Upper Bavarian District Government.

Regarding the cryotome temperature, most tissues were sectioned between − 5 °C and − 25 °C (Caldwell and Caprioli 2005). However, a lower temperature is particularly required for tissues with high fat content. In our study, fresh frozen WAT was cut into sections of 20 μm thickness with a cryotome (CM1950; Leica Microsystems, Wetzlar, Germany) at − 45 °C, to achieve high-quality sections with better integrity and less degradation. Further, all tissue sections were thaw-mounted on indium tin oxide (ITO)-coated glass slides (Bruker Daltonik, Bremen, Germany) pretreated with 1:1 poly-l-lysine (Sigma Aldrich, Munich, Germany) and 0.1% Nonidet P-40 (Sigma Aldrich, Munich, Germany).

### Filter paper application

The filter paper used in this study was purchased from Schleicher and Schuell (Dassel, Germany). It is an ash-free (≤ 0.01%) quantitative paper type with thickness of 0.17 mm and weight of 84 g/m^2^, with wet strength of more than 800 mm water column. The typical particle retention was ca. 2 µm.

When the experimental slides were moved to room temperature of around 20 °C, they were immediately covered separately with a piece of same-sized filter paper in the center, followed by placing a blank glass slide and a flat glass lid in turn right on each filter paper in a vertical direction (Supplementary Fig. 1). This preparation step causes the excess liquid lipids to be removed by the capillary action of the filter paper. A lid and a blank slide weighing around 140 g each were kept in place 1, 10, 30, 60, and 90 s before being removed one by one. The tissue section without the filter paper treatment was compared as the negative normal control. Note that filter paper should strictly move vertically to avoid horizontal movement of the filter paper in contact with tissues, which could damage the tissue samples.

### Matrix spray deposition and MALDI-FTICR imaging MS

Matrix application and MALDI-FTICR imaging MS experiments were performed as previously described (Meding and Walch 2013). Eight layers of matrix solution [10 mg/ml 9-aminoacridine (9-AA) in 70% methanol, purchased from Sigma Aldrich, Munich, Germany] were sprayed using a SunCollect sprayer (Sunchrom, Friedrichsdorf, Germany) at room temperature. The flow rates of the first to eighth layers were 10, 20, 30, 40, 40, 40, 40, and 40 μl/min, respectively. MALDI-FTICR-MSI (solariX 7T; Bruker Daltonics, Bremen, Germany) measurements were performed in negative ionization mode, with a mass range of *m/z* 70–1000 and spatial resolution of 60 μm. All measurements included two nontissue regions as background controls.

### Hematoxylin and eosin (H&E) staining

Following MALDI-FTICR imaging MS, H&E staining was performed using an autostainer (HistoCore SPECTRA ST; Leica Biosystems, Wetzlar, Germany). Further, slides were covered with slips and air-dried in a fume hood until the next scan using a Mirax Desk scanner (Zeiss, Goettingen, Germany) with a ×20 magnification objective lens.

### Data acquisition and analysis

The optical images were acquired with an Axioskop 2 microscope (Zeiss, Goettingen, Germany) before and after applying filter paper or matrix. All images were co-registered and aligned with their corresponding IMS data in FlexImaging v4.0 (Bruker Daltonik, Bremen, Germany). When using FlexImaging, standardization was performed using root mean square to eliminate the difference in intensity between pixels, and reduce the intensity of the background signal, and regions of interest were defined on WAT samples.

The spectrum data were imported into freely available open-source software mMass v5.5.0 (Niedermeyer and Strohalm 2012) and SCiLS Lab v2021a Pro (Bruker Daltonik, Bremen, Germany) for peak picking and ion map visualizations, respectively. The MS spectra were root mean square normalized. The default threshold, which is initially set to select peaks found in at least 1% of the searched spectra, was used to select sample peaks at different periods using SCiLS Lab v2021a Pro (Bruker Daltonik, Bremen, Germany). Metabolites were identified by comparing MS spectra with three datasets (HMDB–v4, LipidMaps–2017-12-12, and SwissLipids–2018-02-02) of the METASPACE annotation platform (Palmer et al. 2017) (https://metaspace2020.eu/). MetaboAnalyst 5.0 (https://www.metaboanalyst.ca/) and KEGG (https://www.genome.jp/kegg/) were subsequently used to perform pathway enrichment analysis. The algorithms used in the MetaboAnalyst platform include a hypergeometric test for overrepresentation analysis and relative-betweenness centrality for pathway topology analysis. The metabolome view was generated according to the* p* from the pathway enrichment analysis and pathway impact values from the pathway topology analysis. Pathway impact is a combination of the centrality and pathway enrichment results. It is calculated by adding up the importance measures of each of the matched metabolites and then dividing by the sum of the importance measures of all metabolites in each pathway.

To confirm the reproducibility of the improved workflow, we also collected and tested three serial WAT sections from one mouse.

## Results and discussion

MALDI-FTICR imaging MS is an emerging method for identifying label-free compounds and determining their spatial localization. The chemical matrix application (Nambiar et al. 2021) and sample preparation steps (Veerasammy et al. 2020) have a significant impact on ionization and the lateral resolution of imaging applications. Given poor matrix deposition caused by a layer of excess liquid lipids on the tissue surface, we aimed to identify a solution to achieve a uniform matrix distribution by reducing excess liquid lipids with filter paper, thereby improving the detection performance of MALDI-FTICR imaging MS in lipid-rich WAT.

**Fig. 1:**
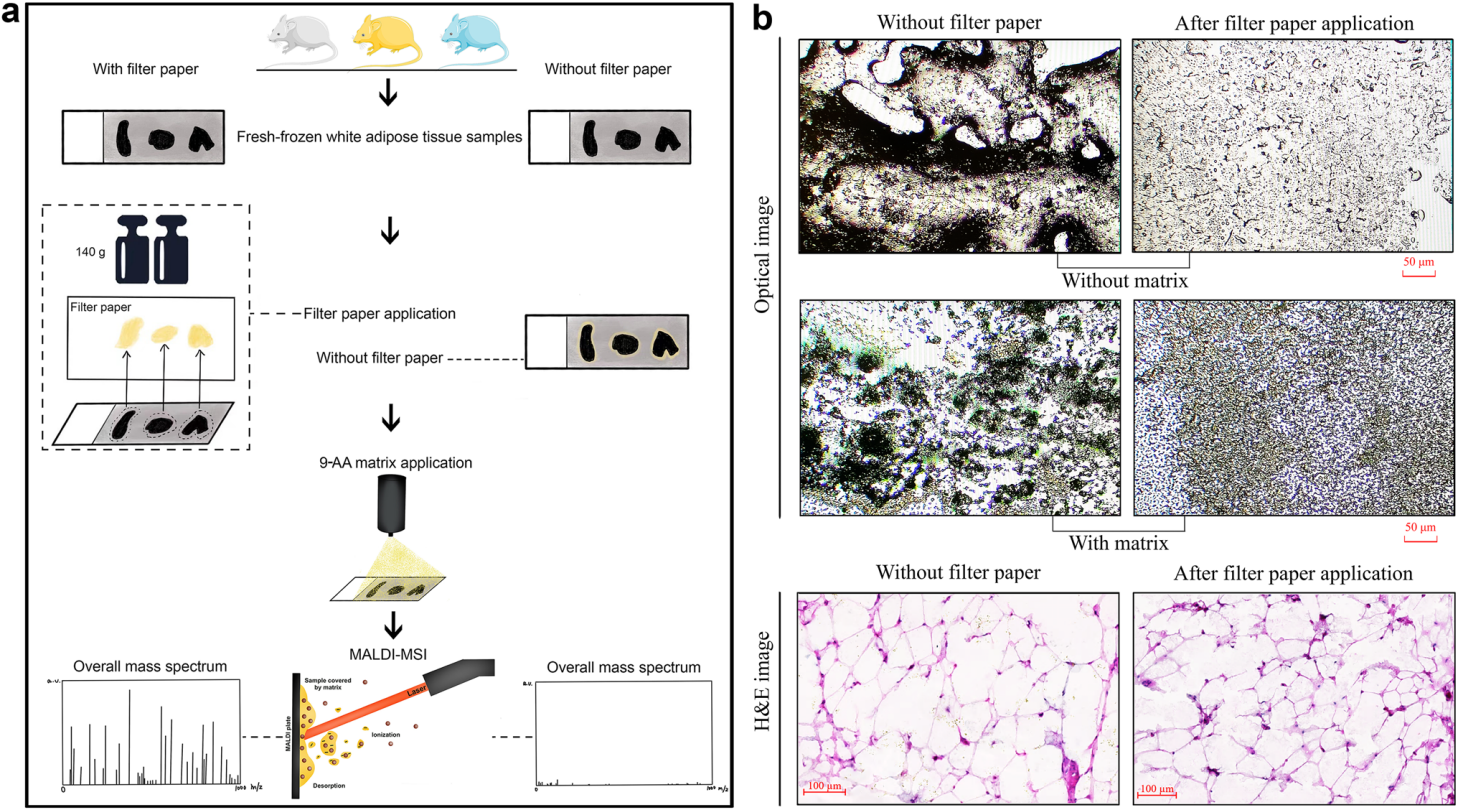
Filter paper removes the liquid lipid layer and achieves better matrix distribution. **a** Scheme of the method and study design. Left workflow: filter paper involved; Right workflow: filter paper-free. **b** Higher-magnification images show that matrix deposited more uniformly in the tissue with filter paper application. The left part is the tissue section without filter paper application, and the right part is the tissue section with filter paper application

To guarantee high comparability in the analysis of results from tissues with and without filter paper application, serial sections of WAT from three mice were collected and thaw-mounted on six ITO-coated slides, followed by consistent filter paper application. The primary goal was to minimize excess liquid lipids and remove the lipid layer formation on the surface of tissue samples so that MALDI matrix deposition could be more uniform than if the matrix was coated directly without filter paper application. The negative control slide followed the workflow without filter paper. Figure 1a depicts the scheme of the whole workflow. As previously stated, it is crucial to deposit matrix uniformly across the tissue section without analyte migration (Collins et al. 2010). In this study, it was achieved by removing the excess liquid lipid layer using filter paper, enabling the detection of more *m/z* species with MALDI-FTICR imaging MS. In Fig. 1b, one can clearly see lipid droplets on the tissue section without filter paper application (left side) and a mixture of liquid lipids and matrix below. In contrast, the matrix on the tissue section with filter paper application (right side) displayed a homogeneous distribution.

**Fig. 2:**
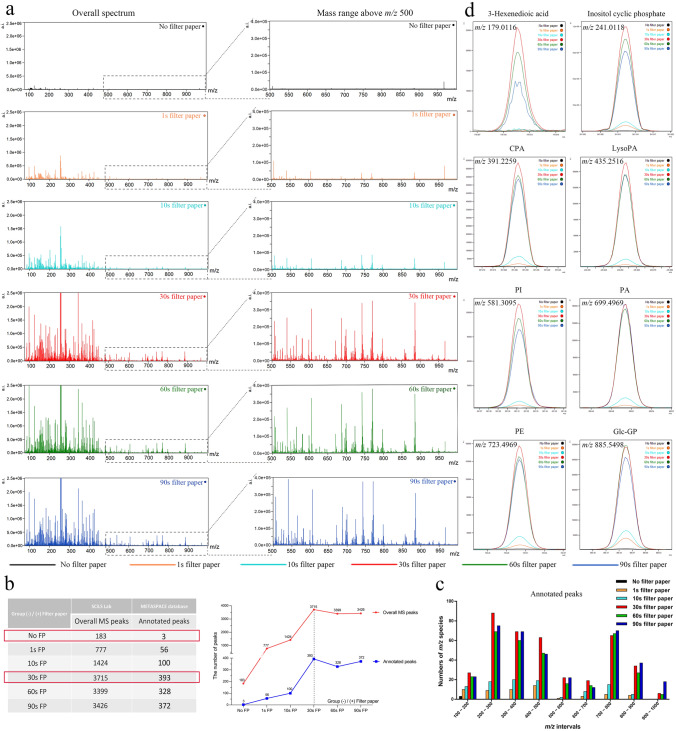
Overall comparison of peaks detected in tissues without and with filter paper application. **a** More peaks were detected in tissues with filter paper application. In particular, the number of peaks at the mass range above *m/z* 500 significantly increased from the sample sections with 30-s filter paper application. **b** 30-s filter paper application enabled detection and annotation of most peaks. **c** The total number of *m/z* species detected (left) and annotated (right) in different *m/z* intervals showed that more lipids at *m/z* range 500–1000 were found in the tissues with 30-s filter paper application (red column). **d** Representative annotated peaks from different mass intervals showed strong intensities in tissues with filter paper application. (*a*.*i*. arbitrary intensity, *CPA* cyclophosphamide, *LysoPA* lysophosphatidic acid, *PA* phosphatidic acid, *PI* phosphatidylinositol, *PE* phosphatidylethanolamine)

Figure 2a shows that, when compared with WAT without filter paper application, the number of *m/z* species detected in WAT with filter paper application increased significantly, particularly after at least 30 s. There were 777, 1424, 3715, 3399, and 3426 *m/z* species found in WAT with 1-, 10-, 30-, 60-, and 90-s filter paper application, respectively, in contrast to WAT without filter paper application, for which only 183 *m/z* species were found. The results from the METASPACE platform showed that 56, 100, 393, 328, and 372 *m/z* species corresponded with annotations in WAT with 1-, 10-, 30-, 60-, and 90-s filter paper application (Supplementary Fig. 3b, Supplementary Table 1), whereas only a few were annotated in WAT without filter paper application (Fig. 2b). Notably, the numbers of *m/z* species both detected and annotated in tissue with 30-s filter paper application were greater than those in tissues with 60- or 90-s filter paper application, indicating that a shorter time is insufficiently effective, whereas a longer time does not increase the number of *m/z* species detected.

Subsequently, we compared the numbers of annotated *m/z* species from each group. On the basis of previous research, MALDI ionization has an unfavorable property of variances in signal intensities caused by matrix crystal heterogeneity, resulting in lowered reproducibility (Edwards and Kennedy 2005). 9-AA, a matrix with few matrix-derived interferences in the low mass range, could significantly improve the sensitivity of MALDI-FTICR imaging MS for analyzing various endogenous metabolites, and is thus recommended as a suitable MALDI matrix for in situ metabolism research (Miura et al. 2010a, 2010b; Yukihira et al. 2010). In the 9-AA negative ion mode of this study, most *m/z* species identified in tissues with filter paper application were at mass ranges of *m/z* 200–500 and 700–900, which are reported to be the ranges of most lipids in 9-AA negative ion mode; in contrast, in tissue without filter paper application, few *m/z* species were only detected at *m/z* 100–200 (Fig. 2c). The results indicated that this approach enhanced the detection of numerous lipids. Although application longer than 30 s appeared to have a negative impact on the number of *m/z* smaller than 700 but a positive impact on larger ones, the increases in numbers for larger *m/z* species after applying filter paper longer were quite low. Since the number of tissue samples was small in the current study, such an observation could be caused by a random effect. The intensity peaks of representative *m/z* species in Fig. 2d showed a significantly better signal-to-noise ratio in the tissues with 30-, 60-, and 90-s filter paper application. These findings indicate that filter paper significantly improved the sensitivity of MALDI-FTICR imaging MS to detect metabolites in lipid-rich WAT.

**Fig. 3:**
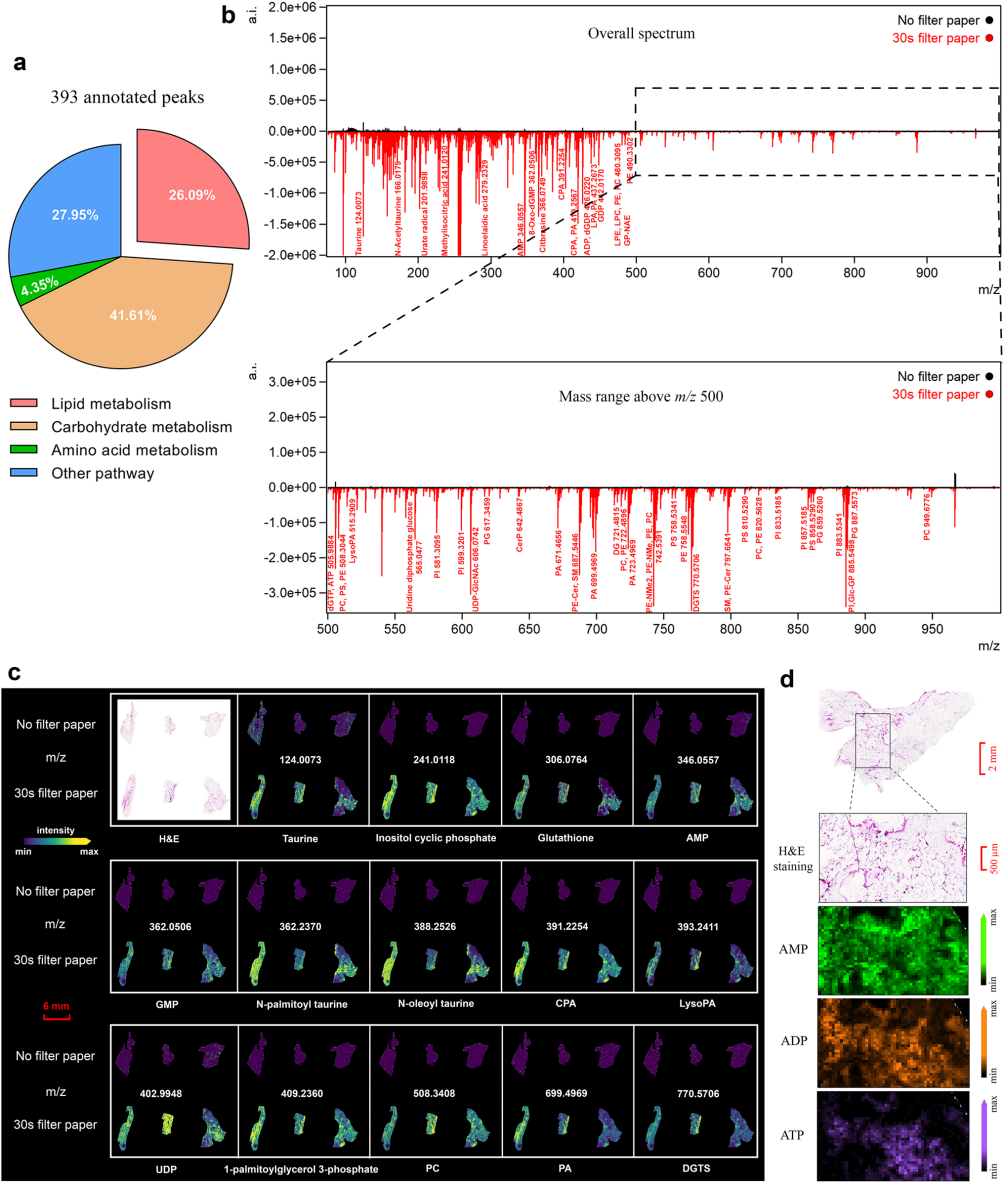
Comparisons between the annotated peaks of tissues treated without and with 30-s filter paper. **a** Pie chart shows the molecules found in tissue with 30-s filter paper application involved in several crucial metabolism pathways. **b** Comparison of annotated peaks from the tissue without application (black), or treated with 30-s filter paper (red), in the overall spectrum (upper), and at mass range above* m/z* 500 (lower). **c** Representative images of endogenous metabolic molecules detected in the tissue using 30-s filter paper showed stronger intensity compared with those in tissue without filter paper application. **d** A higher-magnification image from the WAT section with 30-s filter paper application showed a specific distribution of AMP, ADP, and ATP. (*WAT* white adipose tissue,* a.i.* arbitrary intensity, *PA* phosphatidic acid, *PC* phosphatidylcholine, *PE* phosphatidylethanolamine, *PG* phosphatidylglycerol, *PI* phosphatidylinositol, *PS* phosphatidylserine, *LysoPA* lysophosphatidic acid, *CPA* cyclophosphamide, *AMP* adenosine monophosphate, *ATP* adenosine triphosphate, *ADP* adenosine diphosphate, *GMP* guanosine monophosphate, *UDP* uridine diphosphate glucose, *DGTS* diacylglyceryltrimethylhomoserines)

Further, we conducted more specific comparisons of annotated *m/z* species from WAT with or without 30-s filter paper application, to verify the optimal duration. The molecules identified in tissues with 30-s filter paper application were significantly related to lipid and carbohydrate metabolism, and some of the others belonged to amino acid metabolism (Fig. 3a). The excess liquid lipids that formed on WAT samples severely impaired the spectral quality, such as mass range and signal intensity. Only a few molecules and no lipids were detected in tissue without filter paper application. This contrasts with the result of using 30-s filter paper (Fig. 3b). Several metabolites from the mass range of *m/z* 70–1000 are visualized in Fig. 3c. Applying filter paper for 30 s produced a stronger intensity and consequently better indication of the spatial distribution of these molecules in the WAT section than without using filter paper. By comparing with standard samples, adenosine monophosphate (AMP; *m/z* = 346.0557), adenosine diphosphate (ADP; *m/z* = 426.0221), and adenosine triphosphate (ATP; *m/z* = 505.9884) are displayed at higher magnification as representative metabolites for ion imaging to show differential distributions in tissue with 30-s filter paper application (Fig. 3d). Overall, the best MALDI-FTICR imaging MS performance for detecting metabolites in lipid-rich WAT is achieved when the application time is 30 s.

**Fig. 4:**
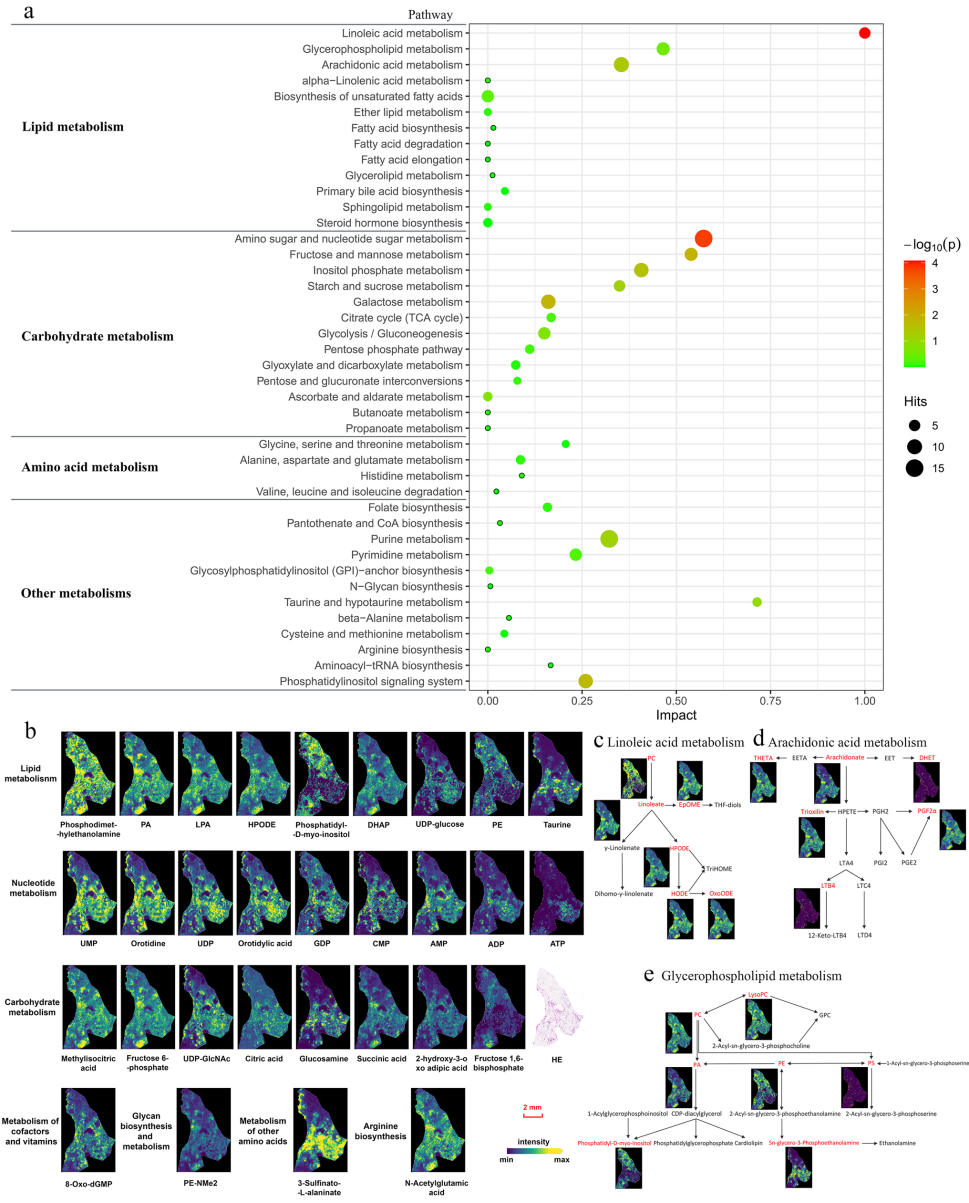
Pathway enrichment analysis of metabolites detected in WAT with 30-s filter paper application. **a** A broad range of metabolites involved in lipid, carbohydrate, amino acid, and other pathways were simultaneously detected in the tissue with 30-s filter paper application. Metabolic pathways are represented as circles according to their metabolic classifications as well as scores from enrichment (vertical axis) and topology analyses (pathway impact, horizontal axis). The color of circles indicates the statistical significance of the overall metabolic changes within the pathway, and circle diameter represents the relative impact of differential metabolites within the pathway as indicated. Pathway impact is a combination of the centrality and pathway enrichment results. It is calculated by adding up the importance measures of each of the matched metabolites and then dividing by the sum of the importance measures of all metabolites in each pathway. **b** Visualization images of representative species in multiple pathways. **c**–**e** show the detected metabolites (red) in the metabolic pathways of linoleic acid, arachidonic acid, and glycerophospholipid, which are top three in lipid metabolism. (*WAT* white adipose tissue, *a*.*i*. arbitrary intensity, *PA* phosphatidic acid, *PC* phosphatidylcholine, *PE* phosphatidylethanolamine, *PG* phosphatidylglycerol, *PI* phosphatidylinositol, *PS* phosphatidylserine, *LysoPA* lysophosphatidic acid, *CPA* cyclophosphamide, *AMP* adenosine monophosphate, *ATP* adenosine triphosphate, *ADP* adenosine diphosphate, *GMP* guanosine monophosphate, *UDP* uridine diphosphate glucose, *DGTS* diacylglyceryltrimethylhomoserines)

To further explore a potential systematic role for metabolite data obtained by MALDI-FTICR imaging MS with filter paper, we proceeded with data on KEGG Mapper and the MetaboAnalyst 5.0 platform. In previous publications, lipid molecules were the primary focus of imaging MS research because they are highly abundant in tissues, and are easily ionized owing to the presence of a polar head (Sugiura et al. 2009; Sun et al. 2008; Fernández-Vega et al. 2020; Popkova et al. 2015; Popkova and Schiller 2017). However, there is little information about other endogenous tissue metabolites in MALDI-FTICR imaging MS, particularly from adipose tissue. In contrast, our study was able to simultaneously detect and visualize multiple endogenous metabolites in WAT sections using 30 s filter paper, including lipid, carbohydrate, amino acid, and vitamin metabolic pathways, and their unique distributions were visualized at 60 μm spatial resolution (Fig. 4a, b). Using 30-s filter paper, multiple metabolites in the pathways of linoleic acid, arachidonic acid, and glycerophospholipid metabolism were successfully detected in WAT (Fig. 4c–e). Linoleic acid is the most abundant fatty acid in adipose tissue and plays an essential role in various physiological and pathophysiological processes in the body, such as pathological pregnancies and human reproduction (Szczuko et al. 2020), microglia inflammation (Tu et al. 2019), as well as breast cancer (Zhou et al. 2016). Most amino sugars are important components of complex molecules, which mainly refer to the various biological macromolecules and microbial secondary metabolites, including antibiotics.

**Fig. 5:**
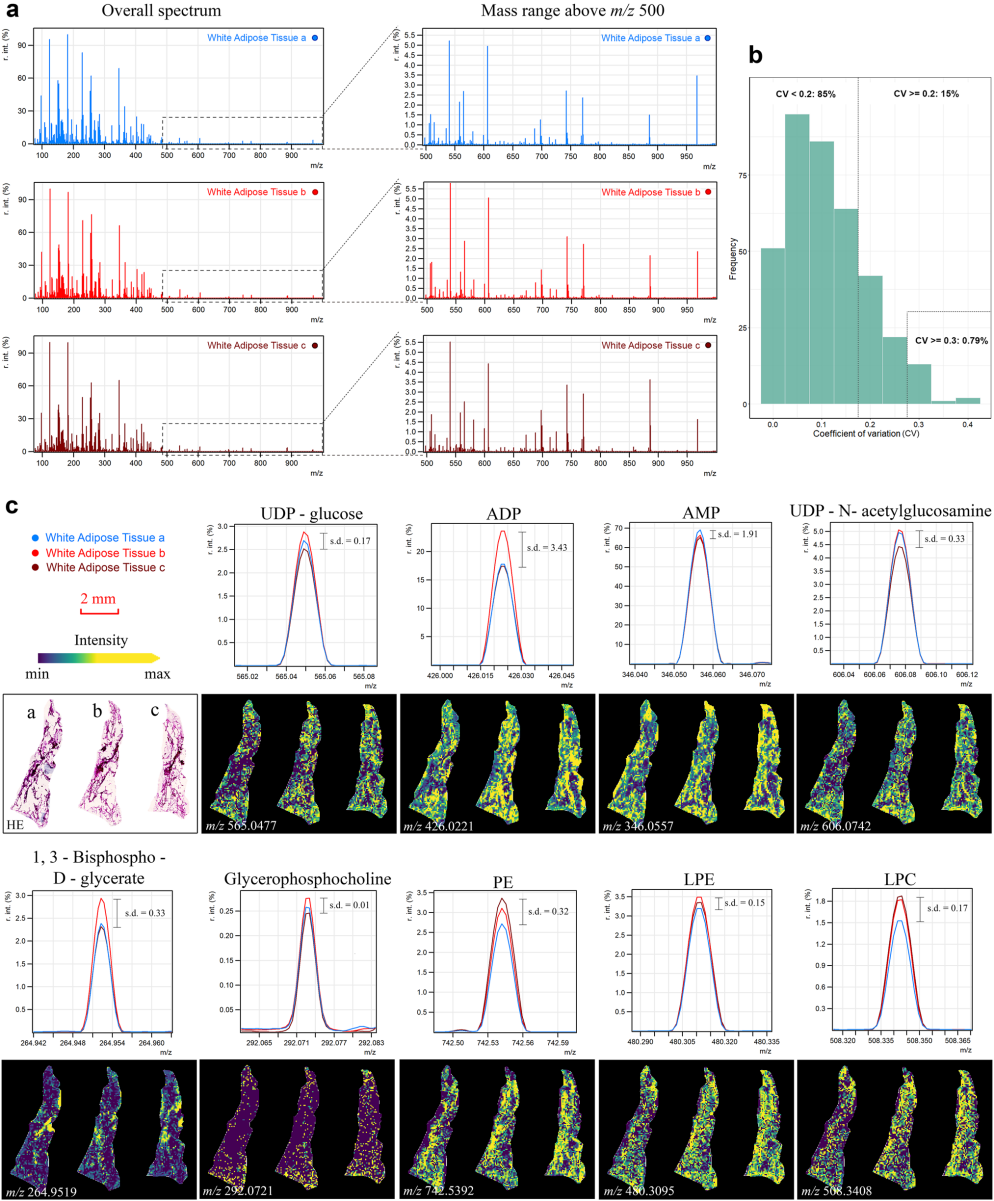
Technical reproducibility of the optimized workflow with 30-s filter paper application. Three serial WAT sections from one mouse were treated with 30-s filter paper and measured at a spatial resolution of 60 µm. **a** Overall (left) spectra and mass range above* m/z* 500 (right) for each triplicate. **b** For the majority of *m/z* species’ intensities in triplicate replications, CVs were below 0.2 and less than 1% of CVs were above 0.3. y-axis, the frequencies of annotated *m/z* species from triplicates; x-axis, the CV values. The dashed line indicates CV = 0.2/0.3 for comparison references. **c** The s.d. of typical endogenous molecules from three WAT sections were calculated and the corresponding optical images (of maximal peak intensities) were visualized. (*WAT* white adipose tissue, *PE* phosphatidylethanolamine, *LPE* lysoPE (lysophosphatidylethanolamine), *LPC* lysoPC (lysophosphatidylcholines), *r*. *int*. (%) relative intensity threshold, *CV* coefficient of variation, *s*.*d*. standard deviation)

Subsequently, to ensure that the optimized workflow with filter paper application is technically reproducible, three serial WAT sections from one mouse were treated with 30-s filter paper, and measured at a spatial resolution of 60 µm. Figure 5a shows that there were no significant differences between replicates when comparing the overall spectra and mass range above *m/z* 500. To determine the reproducibility, we calculated the coefficient of variation (CV) in triplicate tissues. The results are shown in Fig. 5b. For the majority of annotated *m/z* species’ intensities in the triplicate tissues, CVs were below 0.2 (85% *m/z* species) and less than 1% of CVs were above 0.3, which indicates low variation of the test results and good reproducibility of the approach. We also calculated the standard deviation (s.d.) of typically endogenous molecules from three WAT sections and presented them with the optical images together in Fig. 5c. The s.d. values were generally below 0.5. Supplementary Fig. 3a contains more detailed spatial information of molecules and clearly shows their distinctive distributions in the side-by-side adipocytes. Additionally, we selected three regions within the three whole tissue sections, and compared the intensity in these areas by representation in a bar chart; there were no significant variations among the triplicate replications (Supplementary Fig. 3c). Those results further validated that our method has high reproducibility and can be easily applied to any WAT.

Further, we sought to confirm that filter paper does not harm other tissues without the issue of excess liquid lipids. In addition to adipose tissue, the liver is one of the most important central tissues in whole-body energy metabolism. We measured serial liver tissue sections at a spatial resolution of 60 µm to confirm that filter paper has no significant impact on non-lipid-rich tissues. One liver tissue section was tested without filter paper, whereas the other was tested with 30-s filter paper. The comparison of liver tissue spectra with and without filter paper application revealed no significant differences (Supplementary Fig. 2a). Morphological images of tissues with and without matrix and with and without filter paper application are illustrated in Supplementary Fig. 2b. Filter paper did not affect the matrix deposition for liver tissue samples. Supplementary Fig. 2c shows images of the distribution of the typically endogenous molecules detected in both tissues with and without filter paper application as examples. Filter paper has no substantial effect on tissues with low fat content and does not have excess liquid lipid issues when used together.

As the first proof of principle study, the reproducibility issue of this approach at this stage can be further improved. Our approach works well with a balance between time consumed and peaks detected. To obtain the optimal quality and quantity of the results, we recommend researchers pre-test and set the personalized parameters in different working conditions and labs. For example, filter paper application time could differ because of the ambient temperature and properties of the filter paper. The force on the filter paper could also be modified with other flat materials, instead of the lid and blank slide used in our study. Nevertheless, for the purpose of providing a simple and low-cost preparation step to remove the layer of excess liquid lipids, 30-s filter paper application was confirmed to make it attainable and practicable to measure WAT with MALDI imaging MS.

## Conclusion

In this study, we applied filter paper before coating the matrix to overcome the problem of excess liquid lipids on WAT sections when using MALDI-FTICR imaging MS. This is the first report to propose a solution to this limitation. Filter paper application can significantly improve the distribution of matrix and analytical performance of MALDI-FTICR imaging MS for metabolomics research in lipid-rich WAT by removing excess liquid lipids on the surface of tissue sections. On the basis of these findings, the described filter paper application approach for MALDI-FTICR imaging MS in WAT was found to be both cost-effective and highly efficient, requiring only common materials to extend the MSI outcome. The simplicity and reproducibility of our study make the approach practical and readily adaptable by other researchers and wide-ranging WAT projects.

## Supplementary Information

Below is the link to the electronic supplementary material.

Supplementary file1 (PDF 3057 KB)

Supplementary file2 (XLSX 96 KB)

## Data Availability

Data are available from the corresponding author upon request.
