# Supplementary material for: A simple preparation step to remove excess liquid lipids in white adipose tissue enabling improved detection of metabolites via MALDI-FTICR imaging MS
Source: Histochem Cell Biol. 2022 Apr 7;157(5):595–605. doi: 10.1007/s00418-022-02088-y (PMC9114030; doi:10.1007/s00418-022-02088-y)

**Supplementary Figures**

**Supplementary Fig 1 The materials of the filter paper application were placed from bottom to top (corresponding to left to right in the figure)**

When the slides with tissues were moved to room temperature, they were immediately covered separately with a piece of filter paper in the center, followed by placing a blank glass slide and a flat glass lid in turn in the vertical direction. The lid and the blank slide were kept in place for 1, 10, 30, 60, and 90 s before being removed one by one.

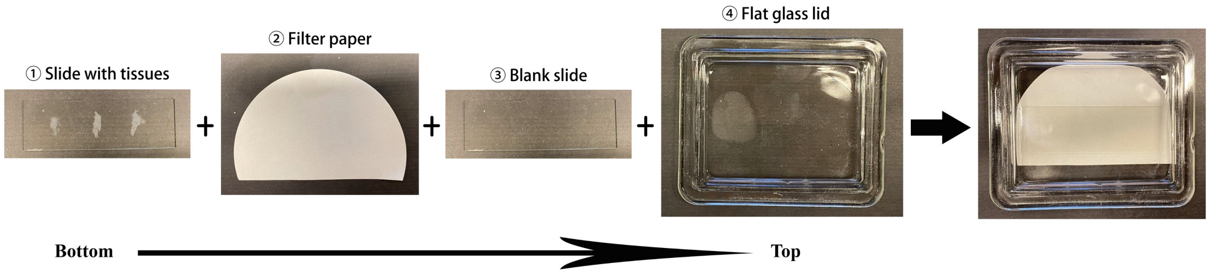

**Supplementary Fig 2 Comparison of the results from liver tissues without or with 30-s filter paper treatment shows that applying filter paper had no significant effect on the tissue without liquid lipid layer on the surface.**

Overall spectra of tissue without filter paper treatment (black) and tissue with 30-s filter paper treatment (red) are shown in **(a)**. Morphological images of tissues with and without matrix or with and without filter paper treatment are shown in **(b)**. Visualizations of representative endogenous m/z species for two tissue sections are shown in **(c)**. (AMP, adenosine monophosphate; ATP, adenosine triphosphate; ADP, adenosine diphosphate; r. int. (%)–relative intensity threshold).

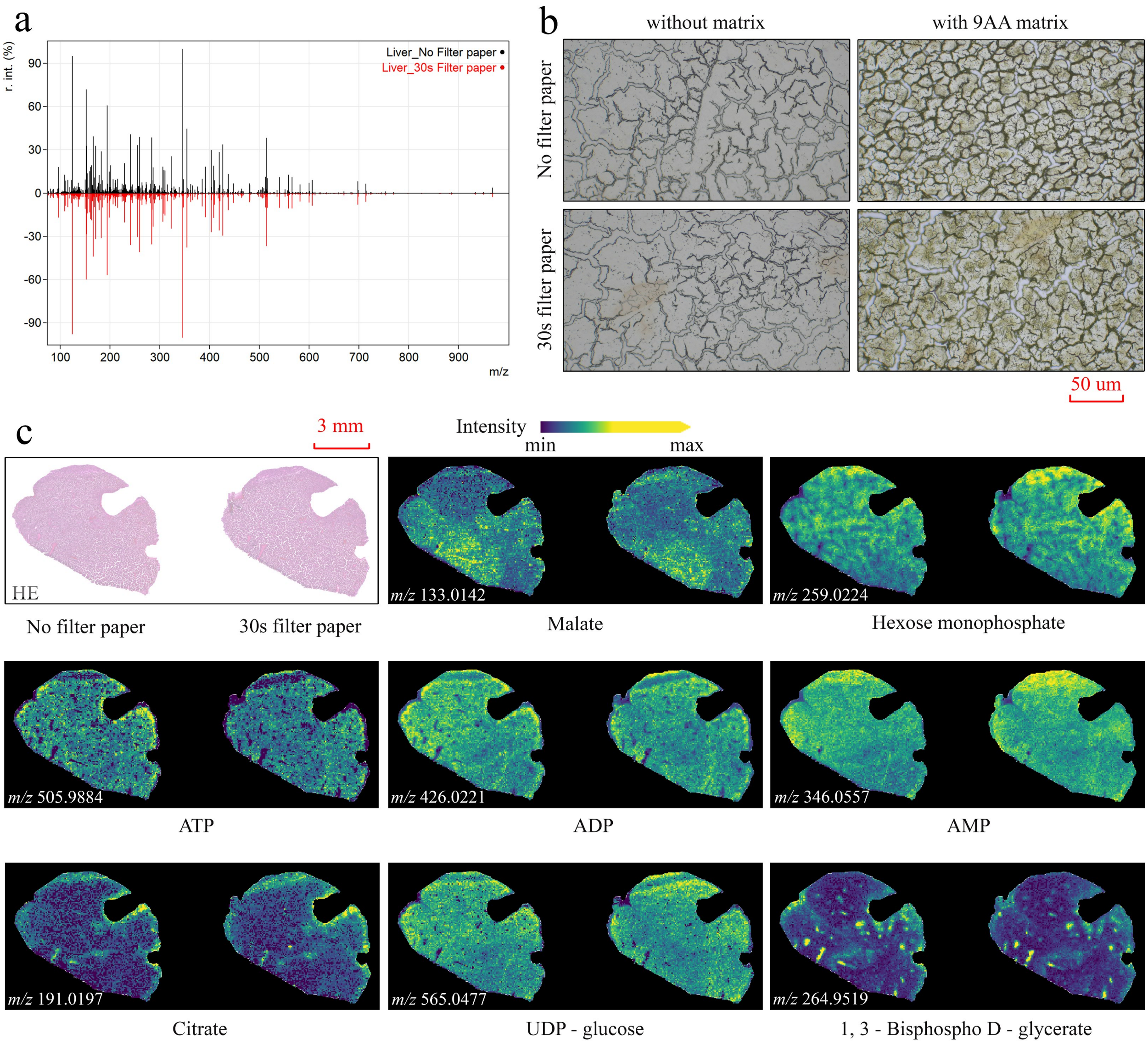

**Supplementary Fig 3 (a)** The distinctive distributions of molecules are clearly visible in the side-by-side adipocytes, which confirmed that the application of filter paper is effective for using MALDI-FTICR imaging MS to study lipid-rich white adipose tissue. **(b)** Venn diagram of annotated peaks shows the number of peaks shared by the different groups. (FP, filter paper; numbers in brackets, the quantities of peaks) **(c)** The intensities in three areas were compared and validated the reproducibility of our approach applying filter paper. (PE, phosphatidylethanolamine; LPE, lysoPE (lysophosphatidylethanolamine); LPC, lysoPC (lysophosphatidylcholines); AMP, adenosine monophosphate; ADP, adenosine diphosphate).

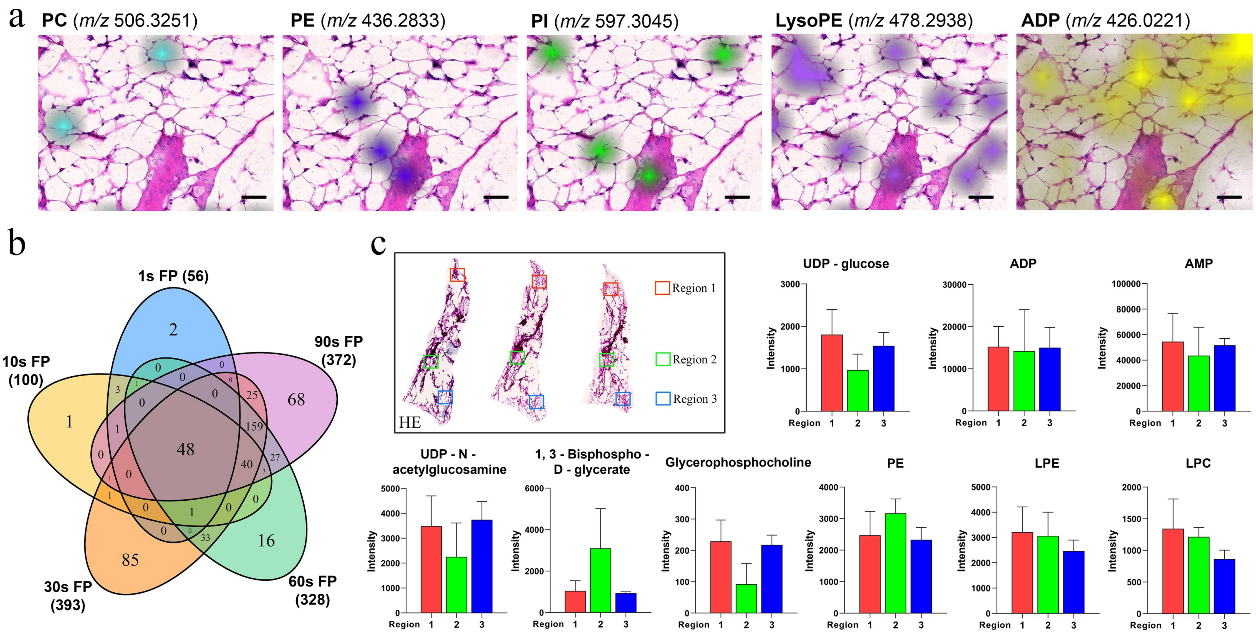

Supplement: Supplementary file 1 — Supplementary file1 (PDF 3057 KB) [file 418_2022_2088_MOESM1_ESM.pdf]
